# Evaluating flow-field and expelled droplets in the mockup dental clinic during the COVID-19 pandemic

**DOI:** 10.1063/5.0048848

**Published:** 2021-04-21

**Authors:** Xiujie Li, Cheuk Ming Mak, Kuen Wai Ma, Hai Ming Wong

**Affiliations:** 1 Department of Building Services Engineering, The Hong Kong Polytechnic University, Hung Hom, Hong Kong, China; 2 Faculty of Dentistry, The University of Hong Kong, Pok Fu Lam, Hong Kong Island, Hong Kong, China

## Abstract

In the setting of widespread severe acute respiratory syndrome coronavirus 2 (SARS-CoV-2) community transmission, reducing the exposure risk on dental professionals and the next patients is the key to reopening dental services in this pandemic environment. The study is motivated by the lack of understanding of the flow-field characteristics and droplet distribution during aerosol-generating procedures. The particle image velocimetry measurements with high temporal and spatial resolutions were performed under ultrasonic scaling in the mockup experimental dental clinic. Compared with other methods focusing on the settled droplet particles, the study focused on the visualization of suspended droplets. From the results of the velocity vector and trajectory map, the high-level contaminated area will be within 1 m from the oral cavity. The vortex structures were identified by the vorticity index. In the surface near the patient's head, a counterclockwise vortex would carry some droplets and contaminate this region. The small droplets circulated in the turbulence cloud and the droplet nuclei generated by dehydration are the two primary sources of suspended particles, which may cause airborne transmission in the dental clinic. About 65%–74% of the droplets in ultrasonic scaling were in the range of 50–180 μm. The research will provide references to the development of the precaution measures to reduce the SARS-CoV-2 exposure risk of dental professionals.

## I. INTRODUCTION

Dental care treatment has been severely affected by the outbreak of coronavirus disease-2019 (COVID-19). In many countries, routine dental treatment was suspended at the beginning of the pandemic, except for emergency treatment in an urgent dental clinic (Kong, 2020). During the period, the aerosol-generating procedures (AGPs) have been avoided unless necessary, resulting in a negative effect on patient care (Long and Corsar, 2020).

In many dental procedures, fluid droplets of various sizes are generated. These droplets and aerosol that harbor infectious virus particles could risk dental professionals and other patients (Ge *et al.*, 2020; Zou *et al.*, 2020). The large droplets fall to the ground and surface quickly because of gravity (Jia *et al.*, 2021; Dbouk and Drikakis, 2020). Therefore, the droplet transmission always occurs in the close distance between the infected individual and the exposed one (Zhang *et al.*, 2013; Bhardwaj and Agrawal, 2020). In comparison, the aerosol and small droplets can stay in the air for a longer time and travel longer distances (Bourouiba, 2020; Ai *et al.*, 2020). The results from other studies have shown that the aerosol and small droplets during the AGPs can travel more than 4 m (Allison *et al.*, 2021). The high-viral loads of severe acute respiratory syndrome coronavirus 2 (SARS-CoV-2) have been detected in the saliva of COVID-19 positive patients (To *et al.*, 2020) and asymptomatic ones (Wyllie *et al.*, 2020). Besides, the proportion of asymptomatic infection in the community is still unknown. Developing a comprehensive understanding of transmission risks during oral health services and assessing the mitigation strategies in dental clinics are the critical components of improving patients' health and reopening dental services in this pandemic environment.

The aerosol and droplet transmission of severe acute respiratory syndrome coronavirus 2 (SARS-CoV-2) has a significant impact on dental therapies since a large number of droplets are generated in the treatment of patients by using rotary, ultrasonic instruments. These aerosol-generating dentistry equipment gain a bit of notoriety, especially during the outbreak of the current COVID-19 (Spagnuolo *et al.*, 2020; Wong *et al.*, 2015). Besides, the occupational noise produced from dental equipment significantly affects the dental professionals and patient's health (Ai *et al.*, 2017; Ma *et al.*, 2017; and Wong *et al.*, 2011). Among the dental instruments, it is commonly believed that the ultrasonic scalers and air-driven high-speed handpieces could produce the greatest aerosols (Bentley *et al.*, 1994).

Most previous research in this area has studied the spatial distribution of droplets or aerosol particles by the microbiological analysis or luminescent tracer. In the microbiological methods, either air sampling (Zemouri *et al.*, 2020) or direct collection onto culture media (Holloman *et al.*, 2015; Rautemaa *et al.*, 2006) were employed to identify the bacterial contamination from the aerosol and droplets. Zemouri *et al.* investigated the microbial composition and spatial distribution of dental aerosol by air sampling, and the highest contamination was found at the patient's chest area (Zemouri *et al.*, 2020). However, only the detected culturable bacteria could be a marker of aerosol and droplet distribution. The luminescent and non-luminescent tracers were also widely used to obtain droplets' spatial distribution and evaluate the exposure risk (Chanpong *et al.*, 2020; Holliday *et al.*, 2021; and Allison *et al.*, 2021). Table I presents an overview of the recent studies on aerosol and droplets in dental clinic. However, the vast majority of these studies focus on the settled particles rather the suspend droplets and flow-field details, which also do not provide sufficient details to accurately estimate the flow velocity and droplets size distribution.

**TABLE I.** Overview of the recent studies on aerosol and droplets in dental clinic.

References	Surgery environment^a^	Methods	Clinical procedures^b^	Investigated parameters^c^
Zemouri *et al.*, 2020	Four dental clinics	Microbiological method (air sampling)	Not mentioned	C_m_; D_s_
Holloman *et al.*, 2015	One dental clinic	Microbiological method (direct culture media)	Ultrasonic scaler	D_s_
Chanpong *et al.*, 2020	One dental clinic	Luminescent tracer	High-speed handpiece, Ultrasonic scaler,	D_s_
			Air-water syringe	
Holliday *et al.*, 2021	Open plan clinic	Luminescent tracer	High-speed handpiece	D_s_; T_s_
Allison *et al.*, 2021	One dental clinic	Luminescent tracer	High-speed handpiece,	D_s_; T_s_
			Ultrasonic scaler, 3-in-1 spray	
Plog *et al.*,2020	Mockup dental clinic	Blacklight shadowgraph	Ultrasonic scaler,	V, d
			High-speed handpiece	

^a^ Dental clinic (single surgery environment), open plan clinic (multiple chairs in one clinical area).

^b^ Not mentioned (depends on individual treatment plan).

^c^ C_m_, microbial composition; D_s_, spatial distribution; T_s_, aerosol settling time; V, velocity of droplets; d, diameter of droplets.

The larger droplets could remain airborne only briefly before settling down to the ground, while the small droplets could evaporate and be suspended as “droplet nuclei” in the air. The behavior is like aerosol, which expands the spatial extent in the enclosed room (Agrawal and Bhardwaj, 2020; Wang *et al.*, 2020). Despite the importance of the spatial distribution of droplets in respiratory disease transmission, there is still a lack of information about the flow-field and persistence of droplets during dental procedures. Specifically, the current lack of research and robust evidence about the flow-field characteristics and size distribution of droplets will become a stumbling block to the reopening of routine dental services. Recently, Plog *et al.* used the blacklight (LED) shadowgraph method to measure the velocity distribution of droplets during the dental procedure (Cavitron scaler), the velocity measurement for 43 μm droplets was just 0.45−0.8 m/s, which was an order lower from the theoretical estimate the of velocity (4.8 m/s) for Cavitron scaler (Plog *et al.*, 2020). A detailed measurement of the velocity characteristics is quite necessary. Besides, since the turbulence cloud emanating from the patient's mouth can spread the virus-containing aerosol and droplets much longer distance, understanding the detailed information of expelled droplets and flow-field is helpful to develop safety precautions. Since most of the previous studies in this area only focused on the settled information, there have never been studied the suspended particle distribution and flow-field characteristics during AGPs. Therefore, the particle image velocimetry (PIV) experiment in this study was proposed to field the research gap, which is the novelty and necessity of this study. The measurement results of this study (like velocity map, vortex identification, and so on) will help to evaluate the specific risks of AGPs and determine the fallow time in dental surgery in the future.

The visualization technology has already shown its advantages in measuring the airflow fields and particle trajectories in a non-intrusive way. In this study, a detailed flow visualization of aerosol and droplets expelled during ultrasonic scaling was obtained by the particle image velocimetry (PIV) technique with laser light and a high-speed CMOS camera. Moreover, this study provided high-quality data for the validation of the CFD codes in dental clinic simulation. In Sec. II, the experimental design and research methods are briefly described. The detailed information of droplets and flow-fields is analyzed in Sec. III. Sections IV and V present the discussion on this study's limitations and the main conclusions, respectively.

## II. RESEARCH METHOD

### A. Dental clinic mockup

**FIG. 1.**
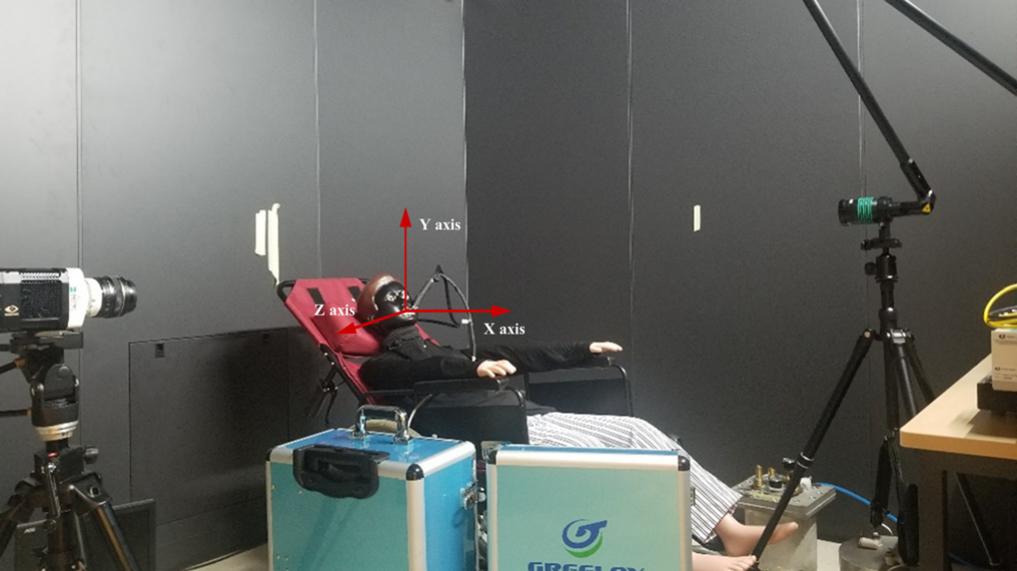
Schematic of the experiment platform.

The mockup experimental dental clinic inside a thermostatic chamber was employed as a platform to conduct a series of measurements. The dimensions of the cabin were 3.6 m length × 2.7 m width × 2.3 m height. The ultrasonic scaler of the portable oral therapy equipment (Greeloy, GU-P 206) was employed in the laboratory experiments. The scaler tip's vibration rate is at 30 kHz, and the cooling water is released by a small opening in the bend of the scaler tip. The water flow rate is about 50 mL/min. The ultrasonic scaler is widely used in dental surgery to remove the calculus deposits from teeth. As for the mechanism of ultrasonic scaling, the high vibration energy from the scaler tip will aerosolize the mixture of coolant water and saliva. A large number of small droplets and aerosol could be expelled from the oral cavity, and thus surrounding airflow will be changed dramatically. The experiment mimicked the situation of a single surgery environment, where the mandibular central incisor of a mannequin or a patient was under ultrasonic scaling. Figure 1 presents the mockup experimental dental clinic used in the laboratory experiments, including the CMOS camera, dental equipment, mannequin, seeding particle generator, and laser. The study was conducted in a stagnant air environment. During the procedure of ultrasonic scaling, the shape of the oral cavity was in an ellipse. The long axis ($D_{L}$) of an ellipse is defined as the *Z*-axis, and the minor axis (coinciding with the sagittal plane) is the *Y*-axis. Three planar slices ($Z/D_{L}=0;\;Z/D_{L}=0.2;Z/D_{L}=0.4)$ were used to measure the flow-field characteristics and droplet distribution.

### B. PIV measurement setup

In this study, the high-power 2D PIV system was employed to investigate the flow-field characteristics and droplet distribution during ultrasonic scaling. The 2D PIV system is primarily composed of a neodymium-doped yttrium aluminum garnet (Nd: YAG) laser with a pulse energy output of 50 mJ at the wavelength of 532 nm, a Dantec SpeedSense Lab 140 CMOS camera with a resolution of 2560 pixel × 1600 pixel, and a synchronizer. In the measurement area, the light sheet with a thickness of 2 mm was formed when the laser beam passes through the cylindrical lens. An automatic transverse gauge could adjust the location of the light-sheet. The CMOS camera with an AF Nikkor 50 mm lens was placed perpendicular to the illuminated plane. The synchronizer is quite critical to obtain a sequence of image pairs by control the time sequence of lasers and a CMOS camera. Di-ethyl-hexyl-sebacat (DEHS) with a mean diameter of appropriate 1 μm was generated by a high-volume liquid seeding generator (10F03) used as tracer particles. There are two atomizer pipes in the seeding generator [high volume liquid (10F03)], and the diameter of each pipeline is around 22 mm. The detailed experimental parameters are shown in Table II.

**TABLE II.** The experimental parameters of the PIV system. DEHS: (Di-ethyl-hexyl-sebacat) with a mean diameter of appropriate 1 μm.

Laser	Laser source	Double cavity Nd: YAG laser
	Laser power	50 mJ/pulse
	Thickness	2 mm
	Time between pulses	68 ms
Camera	Camera model	Dantec 140 CMOS camera
	Resolution	2560 pixel × 1600 pixel
Tracing particles	Seeding generator	High volume liquid (10F03)
	Type	DEHS
	The particle diameter	≈1 μm
	Atomizer pipe	Two pipelines
	The diameter of each pipeline	22 mm
Algorithms	Adaptive PIV with highly accurate sub-pixel interpolation scheme	
	Size of interrogation area (IA)	32 pixel× 32 pixel
	Overlap of interrogation area	50%
	Processing software	Dynamic Studio v5.0
Overall parameters	Sampling frequency	40 Hz
	Sampling quantity	200
	Field of view	0.589 × 0.368 m^2^

The image sequence was acquired by the above PIV system setting with a double frame mode. In order to avoid the test error, the optimizing concentration of tracing particles should be in the range of 10–20 in an interrogation area (Cao *et al.*, 2014), after reaching a relatively steady state. Besides, the laser light sheet suspended in the mockup dental clinic's central could avoid the boundary effect of the wall. The data were assessed by the Dynamic Studio v5.0 software (Dantec Dynamics, New York). First, the background noise was removed by background subtraction. Then, the adaptive PIV method was used to obtain the velocity vector. In comparison with the adaptive correlation method, the adaptive PIV could change the size of the interrogation area (IA) according to the local seeding density and flow gradient. The error about PIV measurement could be divided into systematic errors and statistics errors, which is less than 3.5% in the velocity field (Cao *et al.*, 2014).

## III. RESULTS AND ANALYSIS

### A. Instantaneous flow-field characteristics

**FIG. 2.**
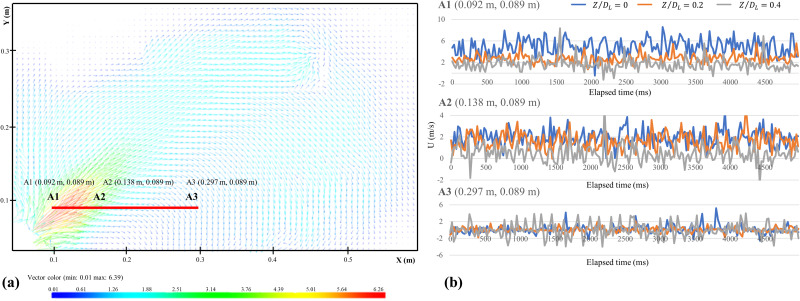
(a) The time-averaged velocity fields in the central axis $\left(Z/D_{L}=0\right)$ and the location of three typical points; (b) The instantaneous U-velocity of three typical points in different planar slices ($Z/D_{L}=0;\;Z/D_{L}=0.2;\;Z/D_{L}=0.4)$.

Figure 2(a) shows the time-averaged velocity vector fields in the central axis $\left(Z/D_{L}=0\right)$. 200 sequential instantaneous velocity fields include $U\;\left(m/s\right)$ and $V\;\left(m/s\right)$ components were used to calculate the velocity vector. The velocity vector fields were generally similar in three planar slices: the airflow issuing from the oral cavity was in an obliquely upward direction. The maximum velocity of the expelled fluid droplet was around 5–6 m/s near the oral cavity. With the increase in the horizontal distance of 0.4 m, it still maintained a speed of nearly 1.2 m/s. When reaching the highest point, the negative-buoyancy droplets began to drop because of gravity, with around 1 m/s initial falling velocity. The falling velocity was reduced to about 0.5 m/s in the lower right corner of the field of view. Although the falling airflow was out of the field of view once they had fallen near the ground area, it was also predictable that the high-level dental contamination range would be within 1 m from the oral cavity. Based on the initial velocity and height, the contaminated area will be within 1 m away from the oral cavity, consistent with the maximum contamination region obtained by the microbiological method (Zemouri *et al.*, 2020). According to Stokes's law, the falling velocity of the droplet scales as its diameter. The decrease in droplets falling velocity could be explained by dehydration, and the detailed analysis about droplets was in Sec. III C.

In Fig. 2(a) three typical points were selected: A1 (0.092, 0.089 m), A2 (0.138, 0.089 m), A3 (0.297, 0.089 m). Point A1 was located near the region of the oral cavity, where the velocity values were the largest. Point A2 was at the boundary of the expelled cloud and point A3 was used to observe droplets' falling patterns. Because the measured expelled droplets by 2D PIV system were spread in *X*-axis and *Y*-axis directions, and the velocity components $U\;\left(m/s\right)$ and $V\;\left(m/s\right)$ were related to the dispersion distance and the sedimentation time, respectively. The U velocity component was selected to present the averaged velocity and velocity fluctuations, as shown in Fig. 2(b). In general, points A2 and A3 had a small fluctuation than point A1. In point A1, the large fluctuation characteristic of velocity is partly because of the different velocities of fluid droplets with different diameters. During the measurement, the fluctuation of velocities in A1 was quite random, and long-term measurement may provide a valuable reference.

The average characteristics of velocity components U and V were calculated in three planar slices, as shown in Table III. When the mandibular central incisor was under ultrasonic scaling, the distribution of generating droplets was not uniform, and the velocity values in the plane ($Z/D_{L}=0.4$) were relatively small. Besides, it should be noted that some particles pass through the laser light sheet ($Z/D_{L}=0.4$), resulting in some errors and high fluctuation.

**TABLE III.** The averaged velocity components in different planar slices.

Planar slice	$Z/D_{L}=0$			$Z/D_{L}=0.2$			$Z/D_{L}=0.4$		
Point	A1	A2	A3	A1	A2	A3	A1	A2	A3
Ave-u (m/s)	4.93	1.95	0.16	2.67	1.62	0.05	1.67	0.44	0.05
Ave-v (m/s)	1.74	0.41	−0.60	2.00	0.48	−0.73	0.95	0.39	−0.01

### B. Statistics results

**FIG. 3.**
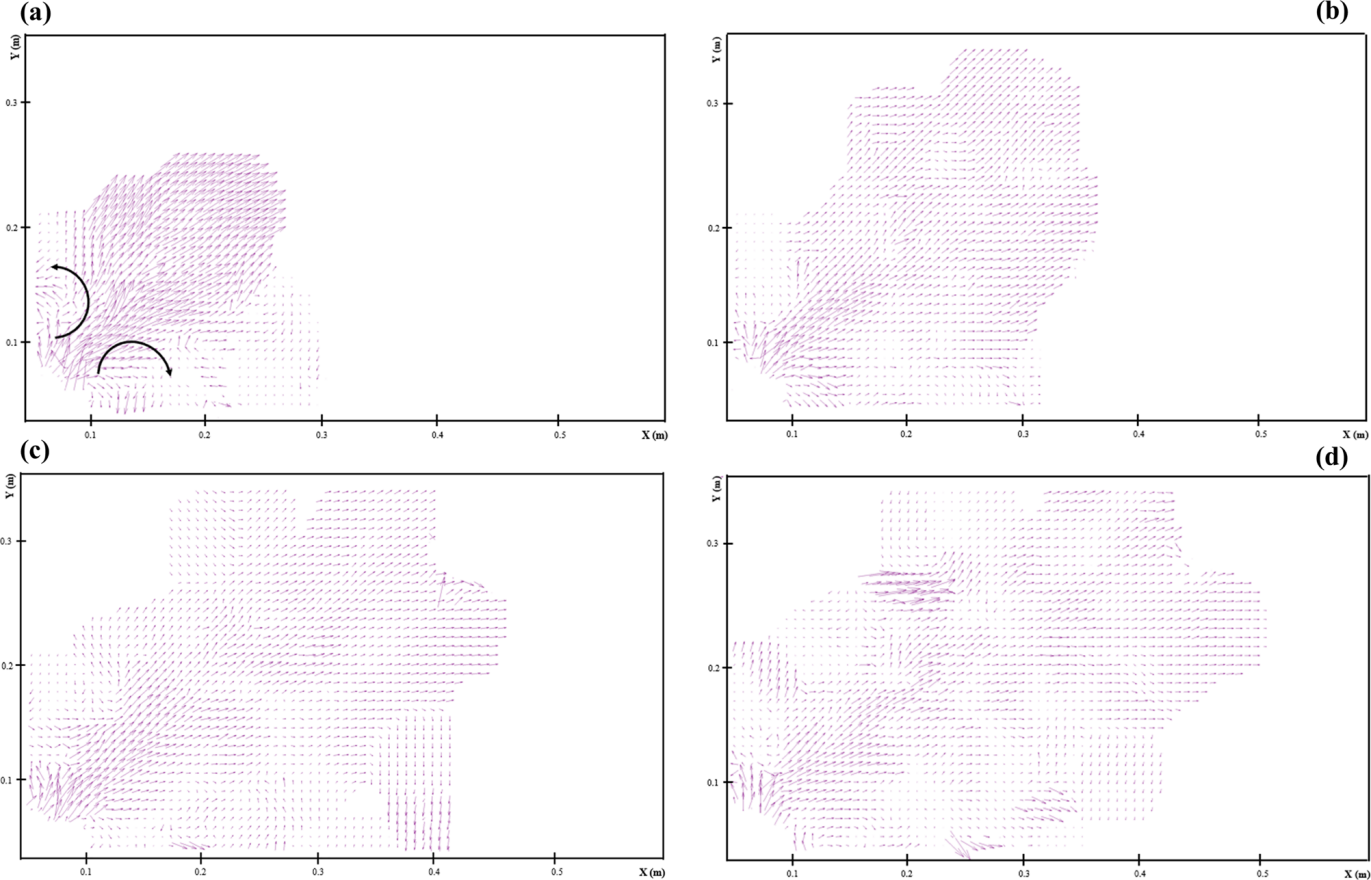
The instantaneous velocity vector fields during the development of ultrasonic scaling: (a) *t*  = 0.02; (b) *t* = 0.05; (c) *t*  = 0.09; and (d) *t*  = 0.24 s.

**FIG. 4.**
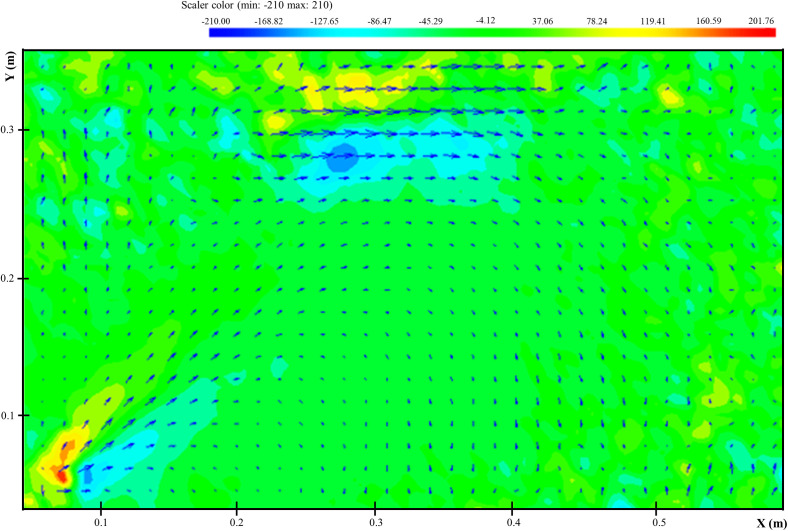
The time-averaged distribution of vorticity.

Figure 3 shows the instantaneous velocity vector fields during the development of ultrasonic scaling. Since the onset of this procedure (*t*  = 0.02 s), a pair of vortexes was presented on the two sides of bell-shaped airflow, and the airflow was relatively stable. Over time, two clear turbulent structures appeared at the top of the field of view. Small droplets were circulated in the turbulence cloud, while the large droplets fall on the ground. Since the vortex plays a critical role in the droplet particle dispersion, the time-averaged vorticity distribution was used to analyze, as shown in Fig. 4. The vorticity can be defined as the following equation:

$$\mathrm{Vorticity}=\frac{{dU}_{y}}{dx}-\frac{{dU}_{x}}{dy}.$$ (1)

The rotational motion of airflow could be described by vorticity ($1/m^{2}$), and the positive and negative values of vorticity represented the counterclockwise and clockwise motion, respectively. Near the oral cavity, the airflow could be divided into two regions by the central axis with vorticities: the negative and positive ones. The positive vorticity meant that the counterclockwise vortex might carry some droplets to the area near the patient's head, and the surface could be contaminated. The precautionary measures should be treated carefully. At the top of the field of view, two neutral vorticities appeared. The negative vorticity was generated, partly because the large droplets circulated in the cloud settled on the high-level contaminated region. The cloud climbs due to the buoyancy effect presented positive vorticity. The small droplets harbored in the cloud can be suspended in the air for a much longer time. That could be a potential source of airborne transmission in the dental clinic.

### C. Droplets distribution

**FIG. 5.**
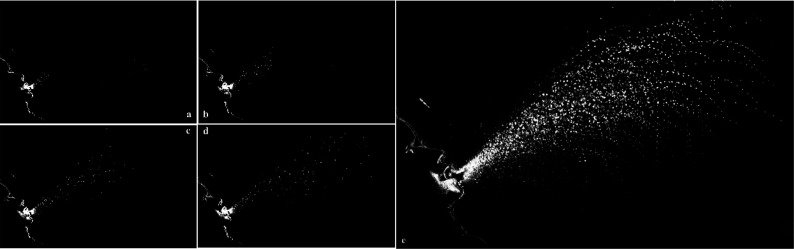
The development of fluid droplets. The image sequence shows multiple snapshots of ultrasonic scaling in the planar slice $Z/D_{L}=0$ (central axis) for the moment (a) 0.02, (b) 0.05, (c) 0.09, and (d) 0.24 s. Figure 5(e) the visualization of expelled fluid droplets and their trajectories. The images are taken from video 1. Multimedia view: https://doi.org/10.1063/5.0048848.110.1063/5.0048848.1

The image sequence recorded by a high-speed CMOS camera in Fig. 5 reveals the dynamics of the expelled liquid droplets phases for the moment (a) 0.02, (b) 0.05, (c) 0.09, and (d) 0.24 s. The images are taken from video 1 (Multimedia view). The onset of visualization was determined by the start of the ultrasonic scaler. The early image in the sequence Fig. 5(a) indicated that the fluid droplets were in high density, and ejected flow was like the cone. In this case, the maximum observed velocity of the expelled fluid droplet is around 6 m/s, consistent with the theoretical values reported by a previous study (Plog *et al.*, 2020). The fluid droplets were transported further over time. It could be seen that the droplets were falling in Fig. 5(d) because of gravity and descended along parabolic-like trajectories. The number of expelled fluid droplets varied widely in the obtained image sequences and significantly affected the dispersion pattern of fluid droplets and even the formulation of precaution measures. It was noteworthy that the variation in the number of droplets is partially due to the laser beam's illuminated region or the vibration frequency of the dental equipment.

When the coolant water is combined with saliva or blood, the bioaerosol, and droplets of various sizes would be generated. In comparison with large droplets quickly falling to the ground, the small droplets can dehydrate as “droplet nuclei” in the air, thereby increasing the exposure risk of dental professionals and next patients. Figure 5(e) shows the visualization of the expelled fluid droplets and their trajectories, and the image is the overlaying of the instant images of the droplets. In the experimental condition: relatively humidity (30%) and temperature (23 °C), the droplets would dehydrate within few seconds. The generated droplet nuclei can be suspended in the air, which may induce airborne transmission of many infectious diseases.

**FIG. 6.**
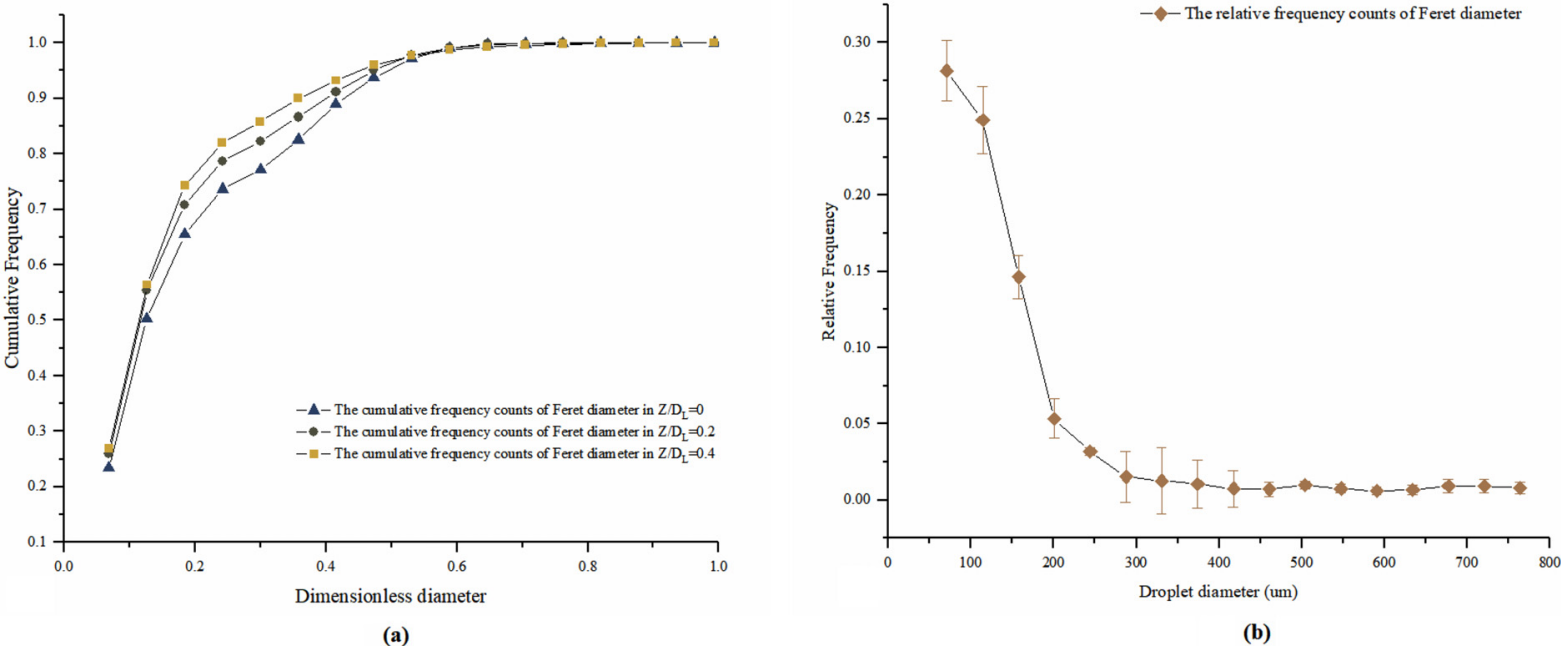
(a) The cumulative frequency with a dimensionless droplet diameter in three planar slices. (b) The population distribution of the diameter of fluid droplets during ultrasonic scaling.

The diameter distribution of the fluid droplets was analyzed by Image J particle counter (National Institutes of Health). After the background noise subtraction, the recorded image sequences were sharpened by turning binary. Selecting the region of interest or defining a mask to cover the facile contour of the subject should be done before carrying out particle counter. According to the diameter threshold, the droplet particles could be identified. The first twenty-one images of obtained image sequences from three planar slices were used to do the droplet analysis. The Feret diameter of droplets was measured before they reached equilibrium states. The cumulative frequency with a dimensionless droplet diameter in three planar slices was presented in Fig. 6(a). The population distribution of the diameter of fluid droplets during ultrasonic scaling was shown in Fig. 6(b) by partially enlarging the photos and keeping the resolution. Due to the collision or neighboring the expelled droplets, the detection algorithm would count as a single droplet, which would over predict the number of large droplets.

The number of identified droplets in the three planar slices varied widely. In the central axis $\;(Z/D_{L}=0)$, the number of droplets was maximized, and the droplets were not evenly distributed in the procedure of ultrasonic scaling. From Fig. 6(a), the cumulative frequency with a dimensionless droplet diameter in three planar slices had a similar tendency. The first three dimensionless diameters accounted for the largest proportion, about 65%–74%. The increasing rate of the middle part of the curve slowed down, and the proportion reached 90% when coming to the ninth dimensionless unit. The proportion of subsequent large particles presented little fluctuations. From Fig. 6(b), the most frequent diameter of the droplet was in the range of 50–180 μm, and the steepest slope was presented between 130 and 180 μm. Due to the overprediction of the detection algorithm in the large droplets, the large droplets at the tail of the distribution curve were detected several times. Thus, it can be concluded that about 65%–74% of the expelled fluid droplets during ultrasonic scaling were in the range of 50–180 μm. It was also notable that the “droplet nuclei” could be generated by the evaporation from the large fluid droplets, and relevant precaution measures should be carefully carried out.

## IV. DISCUSSION

This study visualized the distribution of expelled fluid droplets and the flow-field characteristics during ultrasonic scaling. The PIV provided more reliable and precise results than other methodologies and highlighted the shortcoming of studies that used the microbiological and luminescent tracer methods to obtain the contamination characteristics. The quantitative measurement in velocity distribution and the number of expelled droplets would help evaluate the specific risks of AGPs and develop precaution measures in the dental clinic.

The proportion of asymptomatic infection in the community is still unknown, and the suspicion of infection should be assumed. Although some dental procedures are recommended to be carried out in the negative pressure or airborne isolation rooms, they are not available for all dental clinics. The current precaution measures are insufficient for aerosol management and droplets during AGPs. In many dental clinics, the small droplets circulated in the turbulence cloud and the droplet nuclei generated by dehydration are the two primary sources of suspended particles, which may cause airborne transmission. Although the minimal infection dose of SARS-CoV-2 is not clear, the suspended droplet nuclei and aerosols will increase the exposure risk of the next patients and dental professionals. Some droplet particles carried by the vortex will pollute the surface near the patient's head, and more attention should be paid to this area.

The water widely used as coolant or irrigation liquid could be aerosolized in many dental procedures (e.g., ultrasonic scaler, high-speed handpiece, and 3-in-1 spray). In comparison with the droplet velocity measured near the oral cavity by the blacklight shadowgraph method (Plog *et al.*, 2020), the droplet velocity, ab. 5.0 m/s, measured in this study was much closer to the theoretical estimation (4.8 m/s). Besides, the estimated high-contaminated regions based on falling velocity and trajectory map were in the line with a previous study using microbiological analysis method (Zemouri *et al.*, 2020). About 65%–74% of the diameters of expelled droplets were presented with the range of 50–180 μm, and the droplet nuclei formed by dehydration were observed in the falling trajectories. According to Stokes' law, the terminal falling velocity of droplets is proportional to the square of its diameter. Therefore, the droplet nuclei generated during ultrasonic scaling will persist and slowly descend with the airflow cloud ejected from the oral cavity in the stagnant air environment. At the environmental condition: relatively humidity (30%) and temperature (23 °C) of our experiment, the droplets would dehydrate within few seconds.

A limitation of visualizing the flow-field characteristics and droplet distribution by the image sequence is eliminating many droplets during ultrasonic scaling, although three planar slices have been measured in our study. The flow-field characteristics and droplet distribution have been accurately analyzed from the obtained image sequences, and the droplets out of planes are not considered in this study.

Although the current dental precaution measures (e.g., protective outerwear, protective surgical glasses, and face shields) can effectively prevent the large droplets, developing measures to reduce and isolate the spread of droplet nuclei are also quite critical. It is definitely necessary to do the validation with numerical simulation, and future work will be done in this aspect. Recently, the “fallow time” is recommended in many countries, but there is still a lack of evidence. We also plan to experimentally test the persistence of droplets generated during dental procedures in the near future.

The experiment in this study was carried out in the absence of supply air, representing the neutral conditions. Although the current dental regulations in many countries require dental clinics to have 6 ACH (air change per hour), the ventilation types also play a critical role in the persistence of the droplet nuclei and aerosols. Future research will be conducted in this aspect.

## V. CONCLUSION

In summary, the flow-field characteristics and droplet distribution during ultrasonic scaling were quantitatively obtained by the PIV measurement. The visualization methodology had shown its advantages in obtaining the dynamic pattern of suspended droplets. Three planar slices near the oral cavity have been conducted, and the marked differences have been obtained. Moreover, this study not only provides high-quality data for CFD validation but also helps to develop the precaution measures in dental clinic:

(a) From the instantaneous results, the velocity characteristics of three typical points in different planar slices were analyzed. For the point near the oral cavity (A1), the averaged velocity of this point was highest, about 5.0 m/s, among the three measurement points. The large velocity fluctuation in measurement points is partly because of various droplets in different diameters.
(b) From the statistical results, two obvious vortex structures in the entire flow-fields were observed. A pair of vortices was presented near the oral cavity, which would carry the droplets and contaminate the surface of the patient's head. The second one appeared in the upper area of the field of view. The large droplets would contaminate the area within 1 m from the oral cavity, while some small droplets circulated in the turbulence cloud would be transmitted to much longer distances.
(c) From the visualization of expelled fluid droplets, the most frequent (about 65%–74%) diameter of the droplet was in the range of 50–180 μm. In the falling trajectories, the droplets can dehydrate as “droplet nuclei” in the air. The small droplets circulated in the turbulence cloud and the droplet nuclei generated by evaporation are the two primary sources of suspended particles in the dental clinic.

## Data Availability

The data that support the findings of this study are available from the corresponding author upon reasonable request.
